# An Instrumented Assessment of a Rhythmic Finger Task among Children with Motor Coordination Difficulties

**DOI:** 10.3390/s20164554

**Published:** 2020-08-14

**Authors:** Artal Keinan, Tami Bar-Shalita, Sigal Portnoy

**Affiliations:** Occupational Therapy Department, Sackler Faculty of Medicine, Tel Aviv University, Tel Aviv 6997801, Israel; artalfor@gmail.com (A.K.); tbshalita@post.tau.ac.il (T.B.-S.)

**Keywords:** motor evaluation, low-cost sensorized assessment, thumb-finger test, motor coordination, performance-based assessment

## Abstract

Background: Coordination is crucial for motor function, yet objective clinical evaluations are limited. We therefore developed and tested the reliability and validity of a low-cost sensorized evaluation of a rhythmic finger task. Methods: Children with coordination difficulties (*n* = 24) and typically developing children (*n* = 24) aged from 5 to 7 years performed the Sensorized Finger Sequencing Test (SFST), a finger sequencing test that records the correct sequence, total time, and the standard deviation (SD) of touch time. Additionally, motor performance tests and parents’ reports were applied in order to test the reliability and validity of the SFST. Results: The study group had significantly greater thumb-finger test scores—total time in the dominant hand (*p* = 0.035) and the SD of the touch time in both dominant (*p* = 0.036) and non-dominant (*p* = 0.032) hands. Motor performance tests were not correlated with the SFST. Test–retest reliability in 10 healthy children was found for the SD of touch time in the dominant hand (r = 0.87, *p* = 0.003). Conclusions: The SFST was successful in assessing the movement pattern variability reported in children with motor difficulties. This exploratory study indicates that the low-cost SFST could be utilized as an objective measure for the assessment of proprioception components, which currently are overlooked by standardized motor performance assessments.

## 1. Introduction

The proprioceptive system provides awareness of the body′s movement and posture through receptors found in the muscles, tendons, and joints [1]. Its function is crucial in a child′s development of motor planning and coordination [1,2]. By creating body schema, which is the inner imaging of the body by the brain, proprioception enables effective and controlled movements [3]. Furthermore, smooth performance of sequential activities requires the execution of an appropriate order of action components, as well as the relative timing of these actions. The interrelation between sequencing and timing information improves performance, and possibly serves as a crucial aspect of motor skills [4].

Children with motor coordination difficulties encounter challenges in participating in various activities at school, home, and in the community [5,6], and perform tasks in a less accurate manner compared to typically developing (TD) children. Specifically, these children produce lower scores on tests that involve identifying body postures or executing movements with their eyes closed [7,8] or repeating passive movement of the limbs [9] Additionally, high variability in their gait patterns was reported, suggesting greater difficulty in lower limb control [10]. It is therefore assumed that an altered proprioceptive feedback during movement is one of the underlying mechanisms of motor coordination impairment [11].

A comprehensive evaluation of proprioception is important in order to devise an optimal intervention for children with difficulties in motor coordination [2]. Ayres formed several assessment instruments that included proprioception as part of a conceptual model of sensory integration, e.g., the Sensory Integration and Praxis Test [12], which directly assesses proprioception through joint position and the sensing of movements, but has both psychometric and administrative limitations [2,13]. Other measures that provide indirect information on proprioception are the Sensory Processing Measure (SPM), a caregiver questionnaire [14], and the Comprehensive Observations of Proprioception (COP) [15], which is an observational scale that tests proprioception through behavior and movement patterns. The aforementioned tools provide a subjective observational perception of either parents, teachers, or clinicians, of the child′s proprioceptive ability [2]. Among the clinical observations commonly used as means of assessing proprioception, is the sequential finger tapping test, which requires touching each of the fingertips according to finger order with one′s thumb (opposition), while being blinded to the hands [13]. The sequential finger tapping test scoring relies on the subjective judgment of clinicians and is based on their understanding of proprioceptive function. Accordingly, the test score is dependent on the interpretation of the clinician regarding the observed success in completing the task and/or the effort exerted by the child [13,15,16].

There is, therefore, a paucity of precise and objective tools that evaluate proprioceptive function as part of a standardized clinical assessment [2,17]. Such currently unavailable tools are essential, since treatment could benefit from an objective assessment given that the prevalence of motor coordination difficulties in children is high, e.g., the prevalence of Developmental Coordination Disorder (DCD), a motor coordination difficulty diagnosis, is 6% in children aged 5–11 years [18]. Evidence indicates sensory impairments in DCD, including in the proprioceptive system [19,20,21], yet concerns have been raised regarding the reliability of observer-based assessments of sensation in general [2].

Therefore, in order to accurately assess proprioception, we designed and built an objective low-cost system for quantification of the proprioception components of motor sequencing and timing, the Sensorized Finger Sequencing Test (SFST). We chose to automate the clinical sequential finger tapping test, since this test is widely used as a proprioception marker (e.g., [22,23]). We aimed to examine the test–retest reliability and construct validity (via the known group and discriminant validity procedures) of this newly developed tool.

## 2. Methods

This was a cross-sectional exploratory study. The study was approved by the Institutional Review Board and registered in Clinical Trials.gov (#0038-17-com1; clinicaltrials.gov NCT03285776, respectively). All parents provided a signed informed consent form and all children provided verbal assent for participation in the study.

### 2.1. Participants

Fifty children aged from 5 to 7 years were recruited via a convenience and snowball sampling method, between December 2017 and July 2018. Children who were referred to a child developmental center due to various motor difficulties comprised the study group (*n* = 25), and TD children comprised the control group (*n* = 25). Inclusion criteria were: a final score in the Developmental Coordination Disorder Questionnaire 2007 (DCDQ′07) of ≤46 for the study group and ≥47 for the control group [24]. Exclusion criteria consisted of any neurodevelopmental diagnosis or a major medical disorder. Since the test can be administered to children of both sexes and hand dominancy, we did not exclude children by these factors. Hand dominancy of the child was tested by the hand that the child used to reach to an object placed in the midline of the table. No statistically significant group differences were found in age (tested using the unpaired Student′s *t*-test; Table 1). No statistically significant group differences were found in sex and hand dominancy (tested using the Chi-square test; Table 1). Since there were no differences between the groups in age, sex, and hand dominancy, these factors are not expected to be a confounding factor in our analyses.

### 2.2. Study Tools

The DCDQ′07 was utilized for group placement [24]. This is a standardized caregiver DCD screening questionnaire of motor coordination difficulties for children aged 5–15 years. The questionnaire included 15 items describing motor skills that are rated on a Likert scale of 1–5 (1—not at all like my child; 5—extremely like my child). A final score (the sum of ratings) was calculated and used against the norm cutoffs (for ages 5–7.11 years: a score of 15–46 indicates DCD or suspected DCD, while a score of 47–75 likely does not indicate DCD).

**Figure 1:**
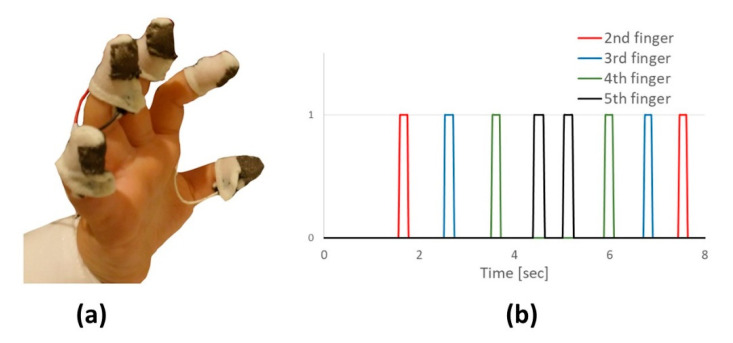
(**a**) The Sensorized Finger Sequencing Test (SFST) was worn on the hand of a subject. A cotton thimble was attached to each finger. A rectangle of conductive fabric was sewn onto the tip of each thimble and connected with wires to a microcontroller that was connected to a computer. (**b**) Example of raw data collected for a healthy subject. Correct finger sequencing is demonstrated over time.

We designed and constructed the SFST used in this study based on the clinical sequential finger tapping test. The SFST was designed to measure proprioception in an accurate and objective manner via five cotton thimbles attached to the fingers (Figure 1a). A rectangle of conductive fabric (MedTex130) was sewn onto the tip of each thimble and connected with wires to an Arduino Mega microcontroller. A code was written for this application, so that when the thumb touched one of the other four fingers, the electronic circle was closed (and remained closed while the touching persisted—see Figure 1b for an example of raw data output). The specified electronic circuit was comprised of two identical resistors, connected in series. One resistor was connected to the 5 v of the Arduino Mega microcontroller on one end, and the other end of the resistor was connected to the thumb. The second resistor was connected to the ground on one end, and the other end of the resistor was connected to one of the four remaining fingers (so there are four sets of resistor pairs, one for each finger). This end was read by a specific analogue channel of the microcontroller and provided 0 v, as long as the finger did not touch the thumb and jumped to 2.5 v when the finger touched the thumb. The code was programed to output ‘0’, as long as the measured voltage of the monitored finger was below 2 v (so that noise is ignored), and ‘1’ when the voltage exceeded 2 v. Since these were simple circuits and algorithms, the outputs were produced with negligible delay. Due to the high conductivity of the fabric (surface resistivity of <1 ohm/sq), a slight touch of the finger and thumb produced a signal. The code registered which finger was currently touching the thumb and the touch duration. The output of the code included the overall test duration, the touch duration (separately for each finger), and the touching sequence. Three measures were extracted for post analysis, separately for each hand: the number of correct finger touches of the sequence before a mistake was detected, total time to perform the test, and the Standard Deviation (SD) of touch time (averaged for the four fingers of each hand). The SD of a measurement captured during a repetitive task, e.g., gait [10] or a paced-finger-tapping task [25] was shown to reflect the variability of the performance.

For construct validity testing using the known group and discriminant validity procedures, the following four assessments were conducted:

- The Movement Assessment Battery for Children-2 (MABC-2) [26]: a standardized assessment of motor skills for children aged 3–16 years. It consists of eight subtests in three domains: manual dexterity (posting coins, threading beads and drawing while following a trail); ball skills (catching and throwing at a target); balance (one leg balance, walking with heels raised and jumping). Subtests for the 3–6 years age group were used. A total test score and a final standard score for each domain were calculated;
- The Beery–Buktenica Developmental Test of Visual-Motor Integration 5th Edition (Beery VMI) [27]: a standard test of visual motor integration for ages in the range of 2–18 years. In this study, two subtests were used: the Visual Motor Integration (VMI), which consists of copying 30 geometric shapes that increase in difficulty, and the Motor Coordination (MC), which comprises drawing in 30 mazes. Both tests were performed with pencil and paper. A final standard score was calculated for each subtest;
- The Sensory Processing Measure (SPM) home form [14]: a standardized caregiver screening questionnaire for children aged 5–12 years. It consists of 75 items that describe behaviors of sensory processing, praxis, and social participation in eight categories. Each item is rated in terms of the frequency of the behavior on a 4-point Likert scale (1-never, 4-always). A standard score is calculated for each category that allows comparison to the norms: typical function in the range of 40–59, some problems 60–69, and definite dysfunction 70–80. We used the following final scores: total sensory systems, body awareness, balance and motion, and planning and ideas;
- The Participation in Childhood Occupations Questionnaire (PICO-Q) [28]. This is a standardized caregiver questionnaire that evaluates participation in activities of children in four main domains: activities of daily living, academic activities, play and leisure, and social functioning. Both versions of the questionnaire were used: for ages 6–10 years (consisting of 30 items) and for ages 4–6 years (consisting of 32 items) (Langer, 2014, personal communication). Each item of both versions is scored on three scales, which together form an overall view of the participation of the child. Specifically, three total scores are calculated: performance difficulty, performance frequency, and activity enjoyment, using a 1–5 Likert scale.

### 2.3. Procedure

**Figure 2:**
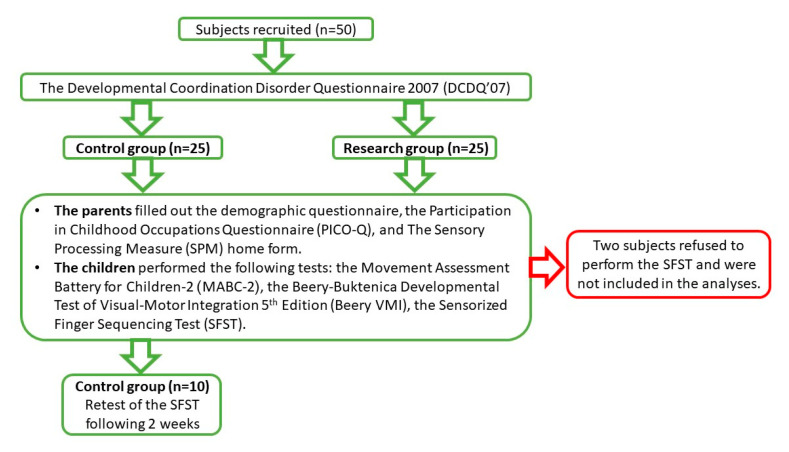
The study outline.

The study outline is depicted in Figure 2. During a one-hour session, the parents completed the questionnaires and the children completed the motor tests and SFST. Before wearing the thimbles, and in order to decrease any testing anxiety and increase their motivation, standard instructions were delivered by the first author (AK) (e.g., “This Spiderman-glove examines how you move your fingers”). The participants were instructed to touch with their thumb on each of their fingertips according to finger order (eight touches with a double touch on the 5th finger). A demonstration was then given. Thereafter, the children were seated at a table with their hands resting behind a small curtain stand, out of their view. They were instructed to perform the sequence once in each hand, starting with their dominant hand. The sequence of the performance tests (MABC, Beery-VMI, and the SFST) was counterbalanced (three sequences) in order to test for bias of attention or fatigue.

For the test–retest, the SFST was administered twice, two weeks apart by 10 control participants, since performance variability characterizes individuals with motor coordination difficulty [10,29].

### 2.4. Data Analysis

The SFST data were analyzed using a LabVIEW code (V2017, National Instruments, Austin, TX, USA), which was written for this study. Three final scores for each hand were used: (i) the number of in-order touches (until a sequence error emerged); (ii) the total test duration; and (iii) the SD of touch duration for all detected touches of each hand.

The statistical analyses were performed using SPSS version 25. One-way analysis of variance (ANOVA) was used to examine the differences between the three sequences of assessments applied. Shapiro–Wilk′s test was used to examine the distribution nature of all variables. Continuous variables were summarized by means and SDs, or medians and inter-range quartiles (IRQ) and groups were compared with *t*-tests for two independent samples or Mann–Whitney tests, according to the data distributions. Categorical variables were summarized according to number and percentage, and groups were compared with Chi-squared tests. Spearman′s or Pearson correlation coefficients were calculated between pairs of continuous variables. All statistical tests were two-sided and tested at a 5% level of significance. Since this was an exploratory study, no alpha correction was applied.

## 3. Results

Since two children refused to wear the SFST, 48 participants (24 in each group) were included in the analyses. The DCDQ′07 final score of the control group was significantly higher than the final score of the study group, with a mean score (SD) of 65.3 (7.2) and 39.5 (4.9), respectively (t(46) = 14.453, *p* < 0.001). All of the study group participants scored below the cutoff that indicates DCD or suspected DCD.

There were no statistically significant differences between the three sequences applied for the following assessment scores: Beery VMI (F(2, 47) = 1.378, *p* = 0.262); MABC-2-ball skills (F(2,47) = 0.861, *p* = 0.429); and total time of the SFST in the dominant hand (F(2, 45) = 1.054, *p* = 0.357). This finding confirms no attention or fatigue bias.

Statistically significant group differences were found in all assessments tested, demonstrating a lower performance in the study group (Table 2).

### 3.1. Construct Validity Testing

**The known group procedure:** The control group performed the trial faster with the dominant hand while demonstrating lower SD touch time in both hands, compared to the study group (Table 3). In addition, although not statistically significant, the control group showed trends towards better performance in the rest of the SFST scores compared to the study group (Table 3).

Discriminant (or divergent) validity: no statistically significant correlations were found between the SFST and the assessments scores of the Beery VMI, MABC-2, SPM and PICO-Q, demonstrating the discriminant type of construct validity, indicating that the SFST tests a different construct than each of these assessments.

### 3.2. Test–Retest Reliability

A statistically significant, positive, and strong correlation was found between the two measurements, only for the SD touch time of the dominant hand (r = 0.87, *p* < 0.01; Table 4).

## 4. Discussion

In this study, we designed and built a precise, low-cost and user-friendly device for the evaluation of proprioception components in children. This is the first study to examine the validity and reliability of the SFST for the assessment of proprioceptive function in children aged in the range of 5–6 years. Importantly, we found that the SFST differentiates between groups, with and without motor coordination difficulties, establishing construct validity through the known group procedure. We further established construct validity through testing discriminant validity—while all assessments used herein tested various types of motor performance, and indeed all found between-group differences, none correlated with the SFST. Thus, it may be suggested that while the SFST measures proprioceptive components that are considered significant for motor performance, it does not assess motor performance, as other assessments do. These findings further emphasize that proprioception and motor performance can be separately quantified, and may confirm the definition of proprioception as the awareness of the spatial and mechanical bodily state [2].

The consistency of the SFST scores was also tested using the test–retest reliability procedure in TD children. Importantly, the measure of the SD touch time (i.e., the consistency of touch times between fingers), which we isolated as a discriminating parameter between children with and without coordination difficulties, was also found to be consistent between test and repeated test for the dominant hand, and thus is a reliable measure of the SFST. However, the total time (i.e., the duration of performing a full cycle in one hand) was reduced in the second measurement. This may suggest a learning effect that elicits improvement in the performance accuracy through implicit learning (i.e., an unintentional and non-conscious learning eliciting behavioral improvement) [30,31]. However, despite the children performing the task faster on the second trial, their performance accuracy was reduced. Such an effect should be further investigated, though we believe that it may be due to the banality of the activity, whereby the task was not novel for the second time yet still required attention.

In this study, completing the SFST took longer for the children with coordination difficulties compared to the control group. Taking longer to complete a task might be a consequence of their difficulty in correcting errors during motor tasks due to their impairment [21]. Interestingly, we found a smaller SD of the touch time in the control group, indicating a higher consistency in touch time between fingers. This demonstrates a movement variation in the study group, and might suggest an explanation for previous reports of the difficulty in maintaining a rhythmic movement pattern in children with motor difficulties [21,32].

This is an exploratory study, testing a newly developed device to ameliorate the proprioception components (i.e., motor sequencing and timing) assessment in children. Yet, some limitations should be considered, such as sample size and potential selection bias based on the recruitment method, missing data, and the number of repetitions in the task performance. Further studies should expand this exploration, comparing eyes open versus closed performance, in order to discern whether the between-group differences stem from deficits in proprioception (and then we expect no between-group differences in the performance with open eyes) or from motor control deficit in keeping a rhythm (and then we expect the between-group differences to exist also when the children are using visual feedback of their finger movements).

## 5. Conclusions

This study lays the foundation for the clinical use and future development and studies of the low-cost SFST, for the clinical assessment of proprioception in particular, and may contribute to the evaluation of motor coordination difficulties in general. Since we found no correlation with the performance-based assessments that are considered to test proprioception as well, the possible potential of the SFST to ameliorate the current evaluation state of performance by contributing an objective measure of proprioception components should be investigated in future research.

## Figures and Tables

**Table 1:** Demographic characteristics of the study group (children who were referred to a child developmental center due to various motor difficulties) and control group. Data are presented as either mean and standard deviation (SD) or number and percent of the study group populations.

		Control Group (*n* = 24)	Study Group (*n* = 24)	
*p*	*t*	Mean ± SD	Mean ± SD	
0.652	−0.454	70.3 ± 7.0	6.9 ± 69.4	Age (months)
***p***	**χ^2^**	**%**	**%**	
0.766	0.356	14 (58.3)	16 (67.0)	Boys
0.724	0.505	20 (83.3)	18 (75.0)	Right handed

**Table 2:** Differences between groups in the motor performance scores. Data are presented as either mean and standard deviation (SD) or median and interquartile range (IQR), according to the distribution of the data.

		Control Group (*n* = 24)		Study Group (*n* = 24)			
*p*	z	IQR	Median	IQR	Median		
<0.001	−4.522	10.7–6.7	25	1.0–9.0	3.5	Total score	MABC−2
<0.001	−4.131	6.0–34.0	9	8.0–1.0	3.5	Manual dexterity	
<0.001	−3.498	75.0–25.0	43.5	5.0–37.0	12.5	Ball skills	
<0.001	−4.226	25.0–63.0	37	5.0–22.7	9	Balance	
<0.001	−4.131	42.0–65.0	58	13.2–43.5	27	VMI	Beery
0.007	−2.707	33.2–72.2	46	9.7–56.7	25	MC	
<0.001	−4.898	47.2–57.0	51	57.0–61.0	63.5	Total	SPM
<0.001	−4.665	51.0–57.0	54	59.0–66.0	64	Balance and motion	
<0.001	−5.326	41.2–52.5	48	57.0–68.5	63	Planning and ideas	
<0.001	−4.507	48.0–56.7	48	57.0–66.0	62	Body awareness	
<0.001	−5.170	1.2–1.4	1.3	1.4–2.6	2.2	Performance level	PICO-Q
0.003	−2.939	3.7–4.5	4.2	4.2–2.9	3.4	Enjoyment level	
	*t*	SD	Mean	SD	Mean		
<0.001	−4.238	0.4	4.2	0.7	3.5	Frequency level	

IQR = Interquartile Range; MABC−2 = Movement Assessment Battery for Children-2; Beery = Beery-Buktenica Developmental Test of Visual-Motor Integration; VMI = Visual Motor Integration; MC = Motor Coordination; SPM = Sensory Processing Measure; PICO-Q = Participation in Childhood Occupations Questionnaire.

**Table 3:** Median and Inter-Quartile Range (IQR) of the Computerized Thumb-Finger Test scores.

			Control Group (*n* = 24)		Study Group (*n* = 24)		
*p*	t/z	IQR	Median	IQR	Median		
0.035	−2.103	9.6–14.2	11.6	11.1–20.3	13.9	Total time (s)	Dominant hand
0.059	−1.887	1.0–5.0	2	0.5–1.5	1	Correct touches	
0.036	−2.094	0.1–0.4	0.1	0.1–0.8	0.4	SD touch time (s) *	
0.075	1.82	3.2 ^a^	10.4 ^a^	4.1 ^a^	12.3 ^a^	Total time (s) ^a^	Non-dominant hand
0.058	−1.899	1.0–5.5	3	1.0–2.0	2	Correct touches	
0.032	−2.142	0.1–0.3	0.12	0.2–0.4	0.2	SD touch time (s) **	

IQR interquartile range. * *n* = 22 in the study group, *n* = 19 in the control (for 7 children data were not recorded). ** *n* = 22 in the DCD study, *n* = 20 in the control group (for 6 children data were not recorded). ^a^ Data are presented as mean and standard deviation (SD).

**Table 4:** Median and interquartile range (IQR) of the Computerized Thumb-Finger Test scores and the Spearman correlations between the two measurement times (test-retest) (*n* = 10).

	2nd Measurement		1st Measurement			
r	IQR	Median	IQR	Median		
−0.08	8.5–10.5	9.3	9.6–14.0	9.8	Total time (s)	Dominant hand
−0.04	1.0–5.0	2.5	2.7–5.2	4.5	Correct touches	
0.87 **	0.1–0.1	0.1	0.1–0.5	0.1 *	SD touch time (s)	
0.06	1.5 ^a^	9.1 ^a^	2.1 ^a^	9.9 ^a^	Total time (s) ^a^	Non-dominant hand
0.18	2.7 ^a^	3.3 ^a^	2.1 ^a^	4.5 ^a^	Correct touches ^a^	
−0.32	0.1–0.2	0.1 ^¥^	0.1–0.2	0.15	SD touch time (s)	

** *p* < 0.01, * *n* = 9 for 1 child, data were not recorded. ^¥^ *n* = 8 for 2 children, data were not recorded. ^a^ Mean and standard deviation (SD) are presented, and Pearson correlation was performed.
